# The impact of circulating 25-hydroxyvitamin D and vitamin D receptor variation on leukemia-lymphoma outcome: Molecular and cytogenetic study

**DOI:** 10.1016/j.sjbs.2023.103882

**Published:** 2023-11-25

**Authors:** Zahraa A. Abdulrazaq, Mushtak T.S. Al-Ouqaili, Nabeel M. Talib

**Affiliations:** a Al-Anbar Health Office, Ramadi Teaching Hospital, Ramadi, Iraq; b University of Anbar, College of Medicine, Department of Microbiology, Ramadi, Iraq; c Al-Anbar Health Office, Anbar Caner Canter, Ramadi, Iraq

**Keywords:** Leukemia, Lymphoma, Vitamin D receptor, PCR-RFLP, Karyotyping

## Abstract

Vitamin D (VD) potentially has a crucial function in the development of cancerous cells. This study aims to detect the role of vitamin D concentration and its receptor polymorphisms as possible prognostic biomarkers in patients with leukemia/lymphoma and further will attempt to detect the presence of the Philadelphia chromosome abnormality in chronic myeloid leukemia (CML). Seventy-five patients, in addition to 50 healthy individuals were included. Three single nucleotide polymorphisms of the vitamin D receptor (FokI, Tru91, and ApaI) were identified via Polymerase Chain Reaction- Fragment Length Polymorphism (PCR-RFLP). Sanger sequencing and karyotyping for all patients has been undertaken. Out of 75 patients, 69 (92.0%) were vitamin D deficient. The homozygous genotype TT of FokI is the most commonly found in non-Hodgkin's lymphoma, while the heterozygous CT is observed markedly in CML, chronic lymphoid leukemia, and Hodgkin's lymphoma. The AC and CC genotypes of ApaI are more frequent in patients with CML, while the AC genotype is the most common in HL. In Tru9I, the GG genotype has a wider distribution in individuals diagnosed with leukemia. The PCR-RFLP and Sanger sequencing techniques together confirmed significant genotype respectively. The Philadelphia chromosome, t (9;22) was found in five (17%) cases with CML. There is a marked relationship between FokI, ApaI, and Tru91 polymorphisms and the chance of developing leukemia. In lymphoma, a significant connection between the polymorphisms of FokI and ApaI is frequently detected. Cytogenetic and molecular testing are essential for detection of CML and monitoring therapy response.

## 1 Introduction

Hematological malignancies (HM) are cancers that affect the cells that produce blood. They are frequently considered to have been formed as a result of improper growth of blood and lymphatic cells throughout different phases of development or differentiation. They comprise nearly 6.5 % of the total number of malignancies globally (Liao et al., 2023). Leukemia and lymphoma are two distinct forms of hematological malignancies (Enawgaw et al., 2021).

Leukemia is defined as an accumulation of abnormal white blood cells, and which accounts for around 3.5 % of all cancer cases (Romo-González et al., 2022). It can be subdivided into four major subgroups in accordance with the kind of cell affected: chronic or acute, and myeloid or lymphoid leukemia (Ali and Salih, 2021). In the vast majority of patients with Chronic Myeloid Leukemia (CML), the cancer is caused by abnormal transmission between chromosomes 9 and 22, creating the chimeric fusion gene BCR-ABL, the so-called Philadelphia (Ph) chromosome (Liao et al., 2023). More than 90 % of cases are caused by the Philadelphia chromosome (Selene et al., 2023). While in CLL cases there are various genetic profiles, TP53 was one of the more common genetic abnormalities recognized (Griffin et al., 2023). Lymphomas are common cancers that can follow an inactive or severe course in the lymphatic system (Watson, 2020). The World Health Organization (WHO) categorizes lymphoid malignancies according to morphology, immunophenotype, genetic discoveries, and tumor cell of origin as to whether they are non-Hodgkin's lymphoma (NHL) or Hodgkin's lymphoma (HL) (Kaya et al., 2022).

VD is a prohormone substance necessary for various different biological functions in the body such as calcium homeostasis, immunological regulation, and cell proliferation (Potre et al., 2023). It also plays a role in reducing lymphocyte and other human-derived malignancy cell proliferations and differentiations. According to previous research, those with low levels (10–30 ng/ml) of 25-hydroxycalciferol (the circulating form of vitamin D) are more likely to develop certain types of malignancies such as solid tumor mass, breast cancer, leukemia (Meta, 2022). Vitamin D acts by adjoining to the VDRs (nuclear receptor) present on many members in the body predominantly localized within the nucleus cells located on chromosome 12q12-q14. (Asghari et al., 2018). Furthermore, the function of VD-mediated activation in carcinogenesis is becoming more widely acknowledged, since numerous researchers have examined the implications of VDR genetic variants in various forms of cancer (Mashhadi et al., 2021).

There are several SNPs that can be detected in the VDR sequence region such as rs2228570 (FokI), located in exon 2, rs7975232 (ApaI), and rs757343 (Tru9I), located in intron 8, that are included in the study. These polymorphisms produce two types of alleles that differ based on the presence or lack of the restriction site, which may be detected by RFLP (Olajide et al., 2023). Many studies have been carried out to determine whether there are genetic variations in a specific region of the vitamin D receptor (VDR) linked to hematological malignancies. Further, the high vitamin D concentration in the blood of individuals with leukemia and lymphoma correlates to better treatment response, longer lifespan, and improved prognosis. Low vitamin D levels are regarded as being a risk factor (Zidan et al., 2019). Therefore, this study aims to detect the role of vitamin D concentration and its receptor polymorphisms as possible prognostic biomarkers in patients with leukemia/lymphoma and further will attempt to detect the presence of the Philadelphia (Ph) chromosome abnormality in CML.

## 2 Patients and methods

### 2.1 Selection of study patients and healthy volunteers

The present study was conducted between September 2022 and May 2023 on 125 individuals submitted to Al-Anbar Cancer Center. They were divided into the following groups: (I) 40 patients with leukemia (30 CML, 10 CLL); (II) 35 patients with lymphoma (20 NHL, 15HL); and (III) 50 healthy subjects as a control (25 with vitamin D deficiency (subjected to RFLP study), and 25 patients who were selected randomly (subjected to ELISA study)). Patients with recent and previous diagnoses of hematological malignancies, either leukemia or lymphoma, were included in the study. The inclusion criteria included both genders (aged > 18 years old) and diagnosed with lymphoma (Hodgkin's and non-Hodgkin's L.) or leukemia (CML and CLL).

Patients with acute leukemia and other malignancies were included in the exclusion criteria. Serum vitamin D was estimated in diagnosed patients and controls. Patients suffering from CML were submitted to karyotyping. The patients and control were presented for the molecular study of the VDR receptor gene’s polymorphism. Routine laboratory examinations, including CBC, were undertaken for all groups.

Ethics statement

The present study complies with the Helsinki Declaration (Ethical Principles for Medical Research Involving Human Subjects). This has been confirmed by the Medical Ethics Committee of the University of Anbar on September 2, 2022, in Ramadi, Iraq (approval number 133).

### 2.2 Serological part of the study

To measure vitamin D concentrations, two milliliters of collected blood was placed in a gel tube and then left for a period of 5–10 min at room temperature, after which it was centrifuged at 1500 rpm for 5 min to separate the serum. All study participants had their vitamin D levels assessed on a fully automated ELISA analyzer (Elisys Uno Human, Germany) by taking 100 µL of serum. In Clinical Practice Guidelines, a level of 25(OH)D less than 10 ng/mL is considered a severe deficiency, while between 10 and 20 and 20–30 ng/mL are considered deficient and insufficient, respectively. Levels of more than 30 ng/mL are defined as sufficient.

### 2.3 Molecular part of the study

#### 2.3.1 Genomic DNA extraction

Two mL of blood was placed in EDTA tubes to investigate VDR polymorphism. The genomic DNA was obtained from collected blood (400 µL) utilizing the SaMag blood DNA extraction kit in conjunction with the SaMag-12 automated nucleic acid extraction equipment (Sacace Biotechnologies, Italy), according to the methods established by Al-Qaysi protocols (Al-Qaysi et al., 2020). Blood DNA extraction involves many sequential stages, including lysis, binding, washing, and elution. The concentration of extracted DNA was determined using a Quantus™ Fluorometer and the QuantiFluor® dsDNA System (Promega, Madison, WI, USA). DNA purity was determined via a UV spectrophotometer, specifically measuring the OD260/OD280 ratio (Kanaan et al., 2023; Al-Ouqaili et al., 2018).

#### 2.3.2 PCR amplification

To amplify DNA fragments, PCR (Polymerase Chain Reaction), ESCO, Riverside, CA, USA, technology was used. The required primers were ordered from the Alpha DNA Company (Canada). The first step was the preparation of stock solution, achieved by dissolving and lyophilizing the primers in nuclease-free water to reach the desired concentration (1.000 pmol/L). The PCR was performed using the Go Taq ® G2 Green Master Mix, acquired from Promega, US. This Master Mix included Taq DNA polymerase, as well as 400 µM each of dATP, dGTP, dCTP, dTTP, and MgCl2 (3 µM), all of which were present in a reaction buffer at pH 8.5. The procedure was conducted using solution (25 μL) comprising 12.5 μL of Master Mix, 1 μL of both forward primer and reverse primer, 4 μL of the target DNA, and 6.5 μL of nuclease-free water. In order to carry out the amplification process of the genes, PCR was conducted using the following conditions. Each gene underwent 35 PCR cycles, the first step of which was initial denaturation (5 min at 95 °C). Subsequently, denaturation was performed at 93 °C for 45 s, followed by annealing for 30 s. at 66 °C for Fok1 and Apa1, and 56 °C for 30 s for Tru91. The extension was then carried out at 72 °C for 45 s. The last step (final extension) was completed at 72 °C for 5 min. The temperature was subsequently maintained at 4 °C. A volume of 5 µL of the PCR product was then introduced into holes made in agarose gel (1.5 %). The electrodes were connected to the power source, and a voltage of 50 V applied for 5 min, followed by an increase to 100 V for 60 min. After that, DNA bands were examined using a UV transilluminator (Vilber Lourmat, Lemont, IL, USA). PCR products with sizes of 740 bp (Apa1), 265 bp (Fok1), and 331 bp (Tru91) were investigated (Table 1).

**Table 1:** The primers used in this study for the detection of VDR genes.

VDR Polymorphism	Primer’s sequence	Products Size(bp)
**Apa1(rs7975232)**	F: 5′CAGAGCATGGACAGGGAGCAAG3′ R:5′GCAACTCCTCATGGCTGAGGTCTCA3′	740
**Fok1(rs2228570)**	F:5′AGCTGGCCCTGGCACTGACTCTGCTCT3′ R: 5′ATGGAAACACCTTGCTTCTTCTCCCTC3	265
**Tru91(rs757343)**	F: 5′AATACTCAGGCTCTGCTCTT3′ R: 5′CATCTCCATTCCTTGAGCCT3′	331

#### 2.3.3 RFLP analysis of the Fok1 (rs2228570) variant

FokI restriction enzyme (Bio Labs-USA) was used to digest the PCR product of the VDR gene (265 bp). The digestion of FokI lasted 3 h at 37 °C, followed by 20 min of inactivation at 65 °C. To examine the enzyme-digested PCR products, 1.5 % agarose gels containing ethidium bromide in 1X TBE buffer solution were electrophoresed at 100 V for a period of 60  min. Digestion of FokI gives C/C 265 bp (homozygote wild type), C/T 265 bp, 169 bp, 96 bp (heterozygote) and T/T 263 bp, 80 bp (homozygote mutant).

#### 2.3.4 RFLP analysis of the Apa1(rs7975232) variant

The ApaI restriction enzyme (Bio Labs-USA) was employed to digest the PCR product of the amplification of the VDR gene (740 bp). After 3 h of ApaI digestion at 37 °C and 20 min of inactivation at 65 °C, the results could be seen on 1.5 % agarose gel dyed with ethidium bromide in 1X TBE buffer solution for 1 h at a voltage of 100 V. Digestion of ApaI gave A/A 740 bp (homozygote mutant), A/C 740, bp, 530 bp, 210 bp (heterozygote), and C/C 530 bp, 210 bp (homozygote wild type).

#### 2.3.5 RFLP analysis of Tru91 (rs757343) variant

The MesI restriction enzyme (Bio Labs, USA) was used to produce the restriction fragment. Tru9I digestion was performed at 37 °C for 3 h followed by inactivation at 65 °C for 20 min before being seen on 1.5 % agarose gel stained with ethidium bromide in 1X TBE buffer solution for 60  min at a voltage of 100 V. Digestion of Tru9I gave G/G 331 bp (homozygote wild type), G/A 331 bp, 178 bp, 153 bp (heterozygote), and A/A 178 bp, 153 bp (homozygote mutant).

#### 2.3.6 Sequencing

The identification of polymorphisms in the research samples used Sanger dideoxynucleoside sequencing technology on the ABI3730XL platform, as provided by Macrogen Corporation (Seoul, South Korea). The sequences were next subjected to analysis using the gene BLAST (Basic Local Alignment Search Tool) tool, accessible via the National Center Biotechnology Information (NCBI) website at https://www.ncbi.nlm.nih.gov/, and sequences were aligned using the Genius software. The aim of the sequencing was to detect the presence or absence of matching between geontypes obtained by PCR-RFLP and those detected in the sequencing.

### 2.4 Cytogenetic part of study (karyotyping)

Patients diagnosed with Chronic Myeloid Leukemia (CML) provided 2 mL blood samples, which were transferred into lithium heparin tubes and immediately sent for culturing and examination to detect chromosomal abnormalities.

Sterile culture tubes were used to transfer about 1,500 µL of blood, which were supplemented with 4 mL of RPMI-1640 medium solution (HiMedia, Mumbai, India). After that, 1 mL of fetal bovine serum (Biowest, Nuaill, France), 200 mL Phytohemagglutinin (Biowest, European), and antibiotics (60 mL ampicillin and 60 mL streptomycin) were added to each centrifuged tube and incubated for 71 h. To inhibit cell growth, 100 mL medication colchicine (Biowest, European) was added to the culture tube, and then after 30 min the cellular suspension underwent centrifugation for 15 min at 1500 rpm. Afterward, the pellet underwent treatment with hypotonic solution (0.075 M KCl). The mixture was well blended, then the tubes were incubated at 37 °C for 15 min. The tubes underwent further centrifugation at 1500 rpm for 15 min. The liquid portion was extracted, and a volume of 7 mL of a chilled, freshly prepared fixative solution (consisting of a 3:1 ratio of methanol to glacial acetic acid) was introduced to the solid residue. The tube underwent a rinsing process, which included being washed three to four times using a new fixative solution for each rinse. After the completion of the centrifugation process, the liquid portion above the sediment was carefully removed. This step was repeated iteratively until a visibly distinct solid pellet was achieved. Subsequently, acquired cells were carefully placed onto pristine slides and subjected to staining using Giemsa, as described by Kanaan et al. (2022) and Khamees and Mushtak (2022).

A stock solution was prepared by combining 2 g of Giemsa stain powder with 100 mL of pure methanol. To achieve rapid staining, a volume of 1 mL (stock solution) was combined with 4 mL (Sorenson's buffer). The status of chromosomal was determined by utilizing the Meta Class Karyotyping device manufactured by Microptics S.L. in Barcelona, Spain, in conjunction with a fluorescence microscope provided by Euromex in Arnhem, Netherlands. The prepared Metaphases and chromosomal were assessed using conventional cytogenetic techniques. The technique of G-banding was used to analyze cytogenetics. A total of twenty metaphases were analyzed in every individual participant. In some instances, the number of metaphases included in the investigation was increased to 50, primarily in situations where abnormalities were identified. The chromosomal aberrations were reported using ISCN (Kanaan et al., 2023).

### 2.5 Data analysis

Variables analysis was conducted using the SPSS software version 22 (IBM Corporation, Armonk, NY, USA). The researchers used an independent *t*-test for comparison between the groups under investigation. A significance level of P < 0.05 was considered to be statistically significant. The statistical software WINPEPI (version 11.63) was used to determine the statistical significance of the P-values obtained by Fisher’s exact test. Additionally, the odds ratio was evaluated using a specific χ^2^ calculation.

## 3 Result

This study was performed on 125 specimens comprised of 75 patients and 50 healthy individuals. The patients were classified according to the type of hematological malignancy into 40 (53 %) patients with leukemia (30 (75 %) CML and 10 (25 %) CLL), in addition to 35 (47 %) patients with lymphoma (15 (43 %) HL and 20 (57 %) NHL).

The age range of study patients diagnosed with CML was 22 to 75 years, with a mean of 41.60 ± 11.746 years. In contrast, the age range for patients with CLL was between 50 and 75 years, with a mean 59.80 ± 7.208 years. The age range of patients diagnosed with lymphoma was observed to be between 18 and 63 years, with a mean of 31.20 ± 10.864 years in HL. In NHL, the age range was between 33 and 65 years, with a mean 51.70 ± 11.859 years. Moreover, this study included a group of healthy individuals (referred to as the control group), and the age range between 18 and 60 years, with a mean of 34.72 ± 12.870 years.

The statistical analysis of WBC (white blood cells), Hb (hemoglobin), and PLT (platelet count) parameters indicates a clear significant difference in patients with CLL in comparison with healthy individuals (P = 0.036) in the WBC values. There was a significant difference (P = 0.056) between platelet count in HL patients with HL and healthy volunteers. All information is recorded in Table S1.

The vitamin D concentration in CML patients had a mean of 13.297 ± 5.3221, while in CLL, HL, and NHL patients the mean VD values were 10.850 ± 4.7535, 9.307 ± 4.1260, and 12.525 ± 7.2608, respectively. Overall, 29 patients (38.6 %) had a severe deficiency (<10 ng/mL), 40 (53.3 %) had a deficiency (10 to 20 ng/mL), five patients (6.6 %) had an insufficiency (10 to 20 ng/mL), and one patient (1.3 %) was in the optimal range (>30 ng/mL) which can be classified as vitamin D sufficiency (Table S2).

According to this study, the level of vitamin D in HM patients was significantly lower compared to the healthy control (p < 0.05). 69 out of 75 patients (92 %) had a vitamin D concentration of < 20 ng/ml. In the controls, five out of 25 individuals (20.0 %) were deficient (≤20 ng/ml). In CLL and HL individual, the ratio of vitamin deficiency was 100 %, with 86.7 % and 90.0 % in the CML and NHL, respectively as represented in Table S3.

### 3.1 The cytogenetic part in patients diagnosed with chronic myelocytic leukemia (CML)

**Fig. 1:**
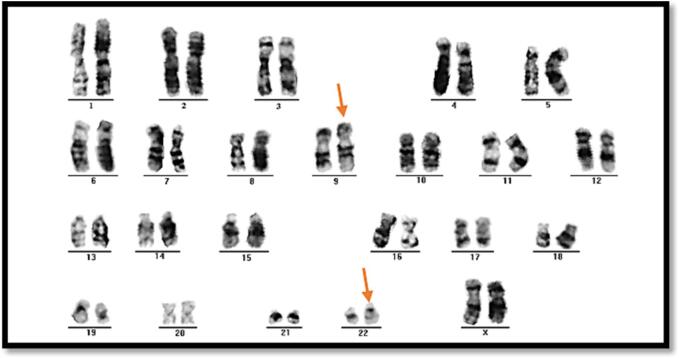
Representative case of an abnormal karyotype of chronic myeloid leukemia patients with t (9; 22).

The karyotyping (chromosomal analysis) to CML patients was carried out for 30 of the study patients. The results of the analysis showed the karyotype was normal in 25 (83 %) of the cases and abnormal in five (17 %) of the cases with CML. Of those with abnormalities, three (60 %) were female and two (40 %) were male, as represented in Table S4. No numerical chromosomal abnormalities were detected; all of these chromosomal abnormalities were structural. The types of structural abnormality cases were due to translocation occurring between chromosome 9 and 22, i.e., Philadelphia chromosome t (9:22) (Fig.1).

### 3.2 The molecular part of the study

#### 3.2.1 Polymerase chain reaction-Restriction fragment length polymorphism (PCR-RFLP)

**Fig. 2:**
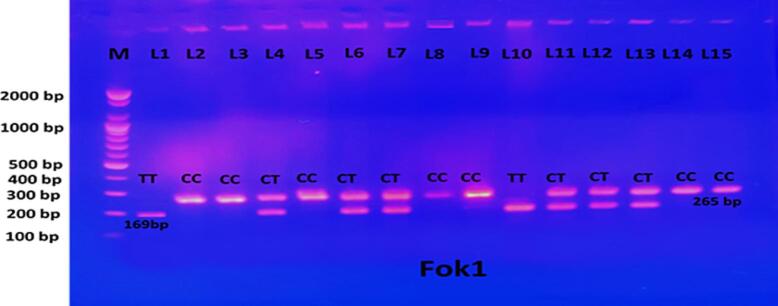
Electrophoto of DNA fragments for polymorphism (FokI) after cutting with FokI enzymes. The results of PCR-RFLP showed homozygote C/C being detected as per the bands at 265 bp, while homozygote T/T was apparent from the bands at 96 bp and 169 bp. Heterozygote C/T was detected with a gene size of 265 bp, 96 bp, and 169 bp. Note that the 96 bp bands ran off the gel.

**Fig. 3:**
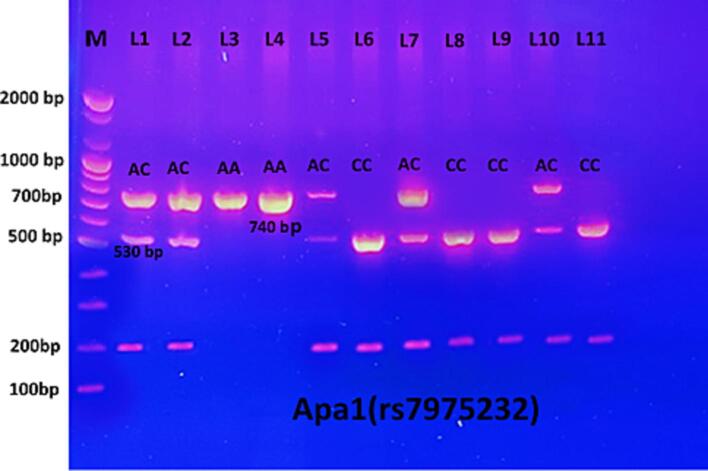
Electrophoto of DNA fragments for polymorphism (ApaI) after cutting with ApaI restriction enzymes. The results of PCR-RFLP showed that Homozygote A/A was detected as per the bands at 740 bp. While homozygote C/C was apparent from the bands at 530 bp and 210 bp. Heterozygote A/C was detected with gene sizes of 740 bp, 530 bp, and 210 bp.

**Fig. 4:**
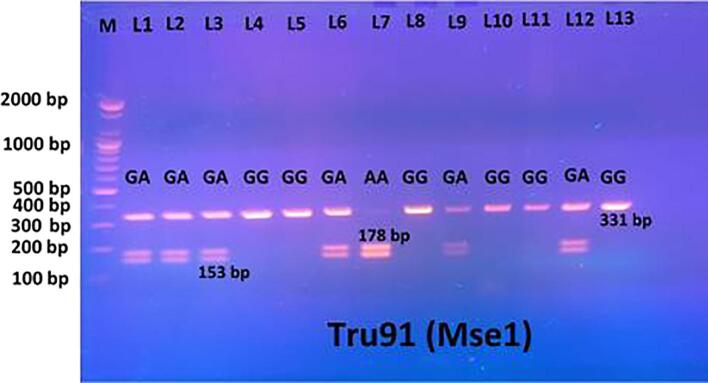
Electrophoto of DNA fragments for polymorphism (Tru9I) after cutting with MesI enzymes. The results of PCR-RFLP showed that Homozygote G/G was detected as per the bands at 331 bp. Homozygote A/A was apparent from the bands at 178 bp and 153 bp. Heterozygote G/A was detected with gene sizes of 331 bp, 178 bp, and 153 bp.

The PCR product of the Fok1 (rs2228570) of the VDR gene on Exon 2 was 265 bp, whilst the Apa1 (rs7975232) and Tru91 (rs757343) genes on Intron 8 were 740 and 331 bp, respectively. The results of amplifying DNA fragments are illustrated in Figures S1-S3. The PCR products were submitted to RFLP and the results reflected in agarose gel electrophoresis. The results of RFLP revealed that three genotypes, (CC, CT, and TT), (AA, AC, and CC), and (GG, GA, and AA) were detected, respectively, as reflected in Fig. 2, Fig. 3, Fig. 4.

#### 3.2.2 VDR gene polymorphism genotype and allelic distribution

The genotypes and allele distribution of vitamin D receptor genes were investigated in this study. The genotype distributions of FokI, ApaI, and Tru9I polymorphisms in both controls and patients with CML were detected, as reported in Table 2. For individuals with CML, the CC genotype of the FokI was apparent in eight cases (26.67 %), with the TT genotype accounting for 6.66 % in two cases, and the CT genotype being the most common in the patient groups, accounting for 66.6 % in 20 cases. This indicates there is a significant role of FokI in CML patients (odds ratio 4.25; CI 95 % [1.34 –13.52]). For the ApaI, genotype, AC was higher in CML than in the control group, accounting for 70.0 % of 21 cases (odds ratio 2.53; the CI 95 % [0.82–7.83], and CC genotypes were detected in six cases (20.0 %) (odds ratio 1.83; CI 95 % [0.40–9.83], revealing an important association with disease, while in Tru9I the GG genotype accounted for 25 cases (83.33 %), indicating this genotype has a positive effect on CML cases (odds ratio 6.36; CI 95 % [1.81–23.33]), the AG genotype accounted for 16.67 % (five cases), and the AA genotype accounted for 0.0 % (0 cases).

**Table 2:** Distributions of vitamin D receptor gene polymorphism in the chronic myeloid leukemia and healthy groups.

Polymorphism	Patients (n = 30)	Controls(n = 25)	P-Value	Odds ratio	CI95%
FokI (rs2228570) genotype					
(CC)	8 (26.67 %)	14 (56.0 %)	0.042	0.29	0.09 to 0.9
(CT)	20 (66.67 %)	8 (32.0 %)	0.011	4.25	1.34 to 13.52
(TT)	2 (6.66 %)	3 (12.0 %)	0.506	0.52	0.06 to 3.85
Alleles’ distribution					
C	36 (60. 0 %)	36 (72.0 %)	0.195	0.58	0.26 to 1.31
T	24 (40.0 %)	14 (28.0 %)	0.195	1.71	0.76 to 3.89
ApaI (rs7975232) genotype					
(AA)	3 (10.0 %)	10 (40.0 %)	0.008	0.17	0.03 to 0.07
(AC)	21 (70.0 %)	12 (48.0 %)	0.08	2.53	0.82 to 7.83
(CC)	6 (20.0 %)	3 (12.0 %)	0.38	1.83	0.40 to 9.83
Alleles’ distribution					
A	16 (80.0 %)	32 (64.0 %)	0.045	0.46	0.21 to 1.0
C	4 (20.0 %)	18 (36.0 %)	0.045	2.17	1.00 to 4.73
Tru9I (rs757343) genotype					
(GG)	25 (83.33 %)	11 (44.0 %)	0.003	6.36	1.81 to 23.33
(AG)	5 (16.67 %)	14 (56.0 %)	0.003	0.16	0.04 to 0.55
(AA)	0 (0.0 %)	0 (0.0 %)	---	---	----
Alleles’ distribution					
G	55 (91.67 %)	36 (72.0 %)	0.008	4.28	1.43 to 14.09
A	5 (8.33 %)	14 (28.0 %)	0.008	0.23	0.07 to 0.7

In CML cases, the T allele (FokI) and G allele (Tru9 I) were correlated with the illness according to the odds ratio, which may possibly be considered a risk allele (odds ratio > 1.0). This may be a reason for increasing the susceptibility to CML. The C allele (ApaI), however, shows no association and is considered protective.

The distribution of the FokI, ApaI, and Tru9I genotypes with the respective allele frequencies and associations was analyzed for CLL and the controls (Table 3). The FokI genotype distribution in CLL was as follows: CC, 60 %; CT, 40 %; and TT, 0 %, while in controls it was CC, 56 %; CT, 32 %; and TT, 12 %. There was a positive correlation between the C allele and the CLL cases compared to the control group (OR = 1.56, CI 95 % [0.45–6.21]). For ApaI, the genotype distribution in CLL was as follows: AA, 60 %; AC, 40 %; and CC, 0 %, while in the controls it was AA, 40 %; AC, 48 %; and CC, 12 %. An important risk factor could be associated with the A allele in CLL cases (OR = 2.25, CI 95 % [0.66–88.0]). The Tru9I genotype distribution in CLL was as follows: GG, 80 %; GA, 20 %; and AA, 0.0 %, while in controls, this was GG, 44 %; GA, 56 %; and AA, 0 %. A clear association was detected between the G allele and CLL cases (OR = 3.5, CI 95 % [0.78–24.53]).

**Table 3:** Distributions of the vitamin D receptor gene polymorphism in the chronic lymphocytic leukemia and healthy groups.

Polymorphism	Patients(n = 10)	Controls(n = 25)	P-Value	Odds ratio	CI95%
FokI (rs2228570) genotype					
(CC)	6 (60.0 %)	14 (56.0 %)	1.00	1.18	0.25 to 5.80
(CT)	4 (40.0 %)	8 (32.0 %)	0.57	1.42	0.28 to 6.70
(TT)	0 (0.0 %)	3 (12.0 %)	0.366	0.00	0.00 to 4.32
Alleles’ distribution					
C	16 (80.0 %)	36 (72.0 %)	0.462	1.56	0.45 to 6.21
T	4 (20.0 %)	14 (28.0 %)	0.462	0.64	0.16 to 2.22
ApaI (rs7975232) genotype					
(AA)	6 (60.0 %)	10 (40.0 %)	0.37	2.25	0.48 to 10.96
(AC)	4 (40.0 %)	12 (48.0 %)	0589	0.72	0.15 to 3.34
(CC)	0 (0.0 %)	3(12.0 %)	0.366	0.00	0.00 to 4. 32
Alleles’ distribution					
A	16 (80.0 %)	32 (64.0 %)	0.208	2.25	0.66 to 88.0
C	4 (20.0 %)	18 (36.0 %)	0.208	0.44	0.11 to 1.50
Tru9I (rs757343) genotype					
(GG)	8 (80.0 %)	11 (44.0 %)	0.046	5.09	0.91 to 39.39
(AG)	2 (20.0 %)	14 (56.0 %)	0.046	0.2	0.03 to 1.10
(AA)	0 (0.0 %)	0 (0.0 %)	---	---	----
Alleles’ distribution					
G	18 (90.0 %)	36 (72.0 %)	0.091	3.5	0.78 to 24.53
A	2 (10.0 %)	14 (28.0 %)	0.091	0.29	0.04 to 1.28

The distribution of allelic and genotypic variations of the VDR gene at the FokI, ApaI, and Tru9I polymorphisms was evaluated in individuals with Hodgkin's lymphoma (HL) and in the control group. The findings of this analysis are summarized in Table 4. The risk allele C (OR = 1.34, 95 % CI = 0.51–3.63) originating from FokI was detected in the genetic study. The CT risk genotype (OR = 1.74, 95 % CI = 0.50–6.05) was also found, indicating that the population carrying this genotype is at an increased risk of developing HL. The ApaI allele C was determined to be significantly correlated with HL (OR = 1.36, 95 % CI = 0.53–3.46). Furthermore, a significant correlation was seen for the genotype AC (*p* = 0.013). No significant difference was observed in Tru9I genotypic frequencies between study patients and the healthy control group (*p* > 0 05). Allele A, derived from the Tru9I gene, was shown to be a risk factor in HL (OR = 1.49, 95 % CI = 0.55–3.95).

**Table 4:** Distributions of the vitamin D receptor gene polymorphism in Hodgkin lymphoma and the healthy control groups.

Polymorphism	Patients(n = 15)	Controls(n = 25)	P-Value	Odds ratio	CI95%
FokI (rs2228570) genotype					
(CC)	11 (55.0 %)	14 (56.0 %)	0.882	0.96	0.29 to 3.23
(CT)	9 (45.0 %)	8 (32.0 %)	0.455	1.74	0.50 to 6.05
(TT)	0 (0.0 %)	3 (12.0 %)	0.161	0.00	0.00 to 2.09
Alleles’ distribution					
C	31 (77.5 %)	36 (72.0 %)	0.55	1.34	0.51 to 3.63
T	9 (22.5 %)	14 (28.0 %)	0.55	0.75	0.28 to 1.98
ApaI (rs7975232) genotype					
(AA)	2 (13.33 %)	10 (40.0 %)	0.122	0.23	0.03 to 1.21
(AC)	13 (86.67 %)	12 (48.0 %)	0.013	7.04	1.36 to 51.83
(CC)	0 (0.0 %)	3 (12.0 %)	0.162	0.00	0.00 to 2.82
Alleles’ distribution					
A	17 (56.67 %)	32 (64.0 %)	0.561	0.74	0.29 to 1.89
C	13 (43.33 %)	18 (36.0 %)	0.561	1.36	0.53 to 3.46
Tru9I (rs757343) genotype					
(GG)	6 (40.0 %)	11 (44.0 %)	0.874	0.85	0.22 to 3.20
(AG)	7 (46.67 %)	14 (56.0 %)	0.636	0.69	0.18 to 2.58
(AA)	2 (13.33 %)	0 (0.0 %)	0.067	---	0.49 to ----
Alleles’ distribution					
G	19 (63.33 %)	36 (72.0 %)	0.391	0.67	0.25 to 1.81
A	11 (36.67 %)	14 (28.0 %)	0.391	1.49	0.55 to 3.95

For both the control and NHL patients, the distribution of the polymorphism (FokI, ApaI, and Tru9I) genotypes and alleles are reported in Table 5. The TT genotype (FokI) was the most common in the patient groups, accounting for 53.34 % in eight cases, indicating there is a relationship with the development of NHL (odds ratio 8.38; CI 95 % [1.68–45.71]).

**Table 5:** Distributions of the vitamin D receptor gene polymorphisms in non-Hodgkin lymphoma and the healthy control groups.

Polymorphism	Patients(n = 20)	Controls(n = 25)	P-Value	Odds ratio	CI95%
FokI (rs2228570) genotype					
(CC)	5 (33.33 %)	14 (56.0 %)	0.153	0.39	0.10 to 1.53
(CT)	2 (13.33 %)	8 (32.0 %)	0.202	0.33	0.04 to 1.75
(TT)	8 (53.34 %)	3 (12.0 %)	0.006	8.38	1.68 to 45.71
Alleles’ distribution					
C	16 (80.0 %)	36 (72.0 %)	0.007	0.26	0.10 to 0.69
T	4 (20.0 %)	14 (28.0 %)	0.007	3.86	1.46 to 10.17
ApaI (rs7975232) genotype					
(AA)	11 (55.0 %)	10 (40.0 %)	0.304	1.83	0.54 to 6.20
(AC)	7 (35.0 %)	12 (48.0 %)	0462	0.58	0.17 to 2.00
(CC)	2 (10.0 %)	3 (12.0 %)	0.821	0.81	0.09 to 6.06
Alleles’ distribution					
A	29 (72.5 %)	32 (64.0 %)	0.434	1.48	0.60 to 3.74
C	11 (27.5 %)	18 (36.0 %)	0.434	0.67	0.27 to 1.67
Tru9I (rs757343) genotype					
(GG)	7 (35.0 %)	11 (44.0 %)	0.659	0. 6**9**	0.20 to 2.36
(AG)	11 (55.07 %)	14 (56.0 %)	0.882	0. 96	0.29 to 3.23
(AA)	2 (10.0 %)	0 (0.0 %)	0.096	---	0.27 to ---
Alleles’ distribution					
G	25 (62.5 %)	36 (72.0 %)	0.314	0.65	0.26 to 1.60
A	15 (37.5 %)	14 (28.0 %)	0.314	1.54	0.62 to 3.80

For allele A (ApaI), the odds ratio was 1.48, suggesting there is relationship with illness based on the odds ratio criteria. This allele may be regarded as a risk allele (odds ratio > 1.0) and may contribute to increased susceptibility to NHL, perhaps playing an etiological role. Furthermore, the odds ratio for variant A (Tru91) was 1.54, suggesting that this allele is related to the rise in the risk ratio.

**Fig. 5:**
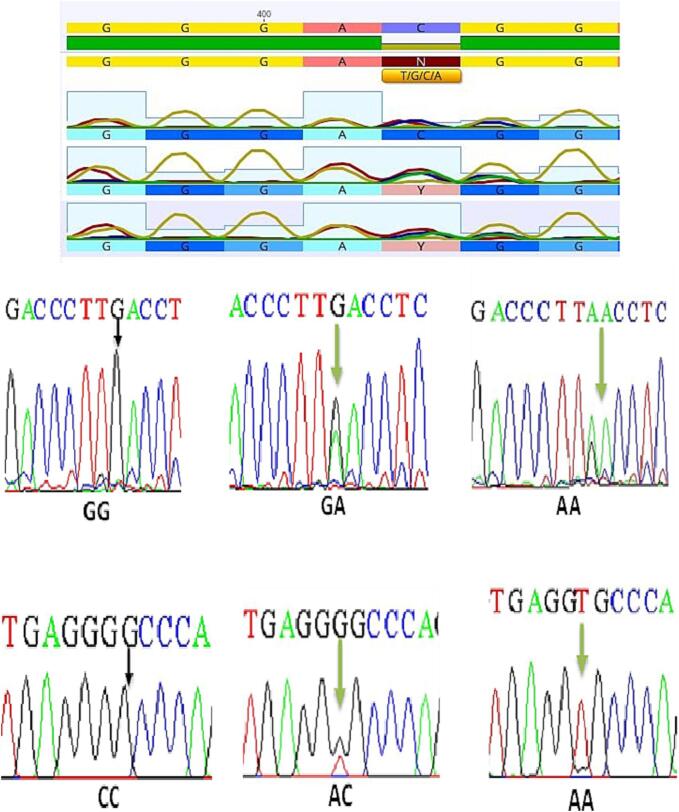
The DNA sequencing for the Fok1 polymorphism (CC-CT-TT) in upper part; Tru91 polymorphism (GG-GA-AA in middle part while the lower part showing Apa1 polymorphism (CC-AC-AA).

PCR-RFLP approach was utilized to detect individual single nucleotide polymorphisms (SNPs). Subsequently, DNA sequencing was undertaken for the specimens previously subjected to RFLP to match the results. After that, the results of sequences were analyzed using NCBI blast online. The genotypes for the Fok1, Tru91, and Apa1 polymorphisms in the chromatogram sequencing are illustrated in Fig. 5.

## 4 Discussion

It is recognized that several epidemiological and clinical studies have shown that low levels of vitamin D increase the chance of developing a variety of cancers. Multiple investigations have shown that vitamin D can normally change various essential cellular processes, including the inhibition of carcinogenesis (Tahir et al., 2023). Although low circulating levels of 25(OH)D may be related to a higher possibility of developing malignancy, the current research shows that vitamin D may enhance outcomes in those diagnosed with cancer (Duffy et al., 2023). Some research suggests that vitamin D supplements can improve the efficiency of therapy, particularly chemosensitivity and drug response. However, some investigations, such as that by Kelly et al., found no significant correlation between deficiency of VD and the reasons for mortality, or survival outcomes (Potre et al., 2023). There are controversial points in the literature regarding the role of vitamin D and vitamin D receptor gene variation in terms of any associated increased risk of developing cancer. Thus, the aim of this study was to determine the role of vitamin D concentration and its receptors using FokI, ApaI, and Tru9I polymorphisms as possible prognostic biomarkers in leukemia/lymphoma.

This study revealed a statistically significant VD concentration in those with HM and those in the healthy control group. Specifically, around 92.0 % of HM patients exhibited a deficiency in vitamin D, while just 1 % had sufficient amounts of VD, that is, more than 30 ng/ml. However, the association between deficiencies in VD appears to be stronger in specific leukemoid and lymphoid malignancies, including CML, CLL, HL, and NHL. Moreover, the level of VD was connected to an elevated risk of hematologic malignancies. The elevation of the concentration of 25(OH)VD3 in the blood has been shown to contribute to both the initiation and advancement of tumor growth (Dehghani et al., 2021). Data from some research shows that 1,25(OH)2D3 reduced the proliferation of abnormal cells and activated cell differentiation, and further stimulated cell death in variety of cell types expressing VDR. It was also found that 1,25(OH)2D3 may have anticancer activity against leukemias (Rui et al., 2020).

The present investigation revealed a positive association between decreasing levels of VD and patients diagnosed with CLL and HL, with a prevalence of 100 %, while CML and NHL showed prevalences of 86.7 % and 90.0 %, respectively. The present study examined the impact of VD and its correlation with prognostic markers in HL and CLL malignancies. The findings revealed a substantial decrease in VD levels among CLL patients, suggesting a potential relationship with advanced stages of the disease (Dehghani et al., 2021). Previous research suggested a connection between the molecular response to TKI therapy and vitamin D levels. The published literature regularly reports that vitamin D insufficiencies correspond to poorer treatment responses in CML patients and that supplementing people with such deficiencies can improve their responses (Mulhim et al., 2021). However, many studies found the levels of VD altered the molecular response in patients, and propose that vitamin D levels might be tested in individuals with HM, as well as administering vitamin D replacement drugs to patients who have a deficiency (Gediz et al., 2020).

In our study, we examined the role of VDR in leukemia and lymphoma patients with a focus on the impact of the SNPs ApaI, Tru9I, and FokI in the VDR gene. The results obtained from the PCR-RFLP analysis indicate that the homozygous genotype CC of Fok1 (rs2228570) in the VDR gene was the predominant genotype seen in CLL. The heterozygous genotype CT was shown to be the most prevalent among individuals diagnosed with CML and NHL; for HL, it was observed that the homozygous genotype TT has a higher prevalence. In addition, it has been shown that allele C is a causative factor that can be linked to illness development in CLL and HL; in contrast, allele T is implicated in CML. The NHL allele T exhibits a much greater prevalence in the control group, suggesting that it may be a protective factor, though this is contingent upon the odds ratio.

The prevalence of the CC (homozygous genotype) and AC (heterozygous genotype) of Apa1 (rs7975232) is higher in individuals diagnosed with chronic myeloid leukemia (CML) compared to the control group of healthy individuals. The heterozygous genotype AC had the highest prevalence among individuals diagnosed with HL. The homozygous genotype AA exhibited a statistical increase in CLL and NHL. Moreover, it was shown that allele C had a statistically significant positive correlation with the occurrence of the illness in CML and HL. In contrast, it was shown that allele A exhibited a significant association with the occurrence of the illness in both CLL and NHL, as indicated by the odds ratio. These results correspond with those of Pezeshki et al., who found that the CT genotype (Fok1) was significantly associated with CML, while the T allele could be considered a prognostic agent (Pezeshki et al., 2018). However, it should be noted that the findings of this current study do not agree with previous investigations, which indicated that the CC genotype, Fok1, demonstrated lower prevalence in the chronic lymphocytic leukemia (CLL) group in comparison with a healthy control group (Rajab et al., 2015). Several results have also been reported in hematologic malignancy investigations. However, some studies have found a link between Fok1 polymorphism and an increased risk of leukemia, but no links were found between Fok1 polymorphism and lymphoma (Asghari et al., 2018).

Our results indicate a positive relationship between Fok1 and Apa1 polymorphisms in lymphoma (HL and NHL) patients, findings that contradict a previous study that was carried out to evaluate the vitamin D receptor genotype and allele distribution in NHL, and which showed no clear connection between NHL and FokI or ApaI polymorphisms (Mashhadi et al., 2021). Another study on patients with HL found no relationship between the risk of developing HL and the Fok1 and Apa1 variants (Tekgündüz et al., 2017).

For Tru91 polymorphism in the VDR gene, the homozygous genotype GG was the most common in cases of CML and CLL, and which had no impact on lymphoma (HL and NHL) illness. Furthermore, allele G is considered to be amongst the reasons for the development of the disease depending on the odds ratio, so the genotype GG (Tru9I) is related to an increased chance of the progression of leukemia (CML and CLL). Very few studies have been conducted that examine the relationship between Tru9I and cancer, such as colorectal carcinoma, breast cancer, and prostate cancer. One previous study showed that genotype AA can be correlated with an increased risk of breast cancer but, however, has a negative role in prostate cancer (Iqbal et al., 2018). We have hypothesized that VDR gene polymorphism may be an important factor in the risk of disease and the response to treatment in patients with HM.

Thirty CML patients participated in this study. According to cytogenetic characteristics, such as the presence or the absence of a translocation between chromosomes 9 and 22, they are differentiated. The results were interpreted as meaning that the ratio of patients with Philadelphia translocation t (9;22) was 17.0 % (No. 5/30), where those patients with positive results were reached as soon as possible, before treatment (one case) and between 1 and 4 months after starting therapy (other four cases), which is consistent with a previous study in which 39 patients were subject to cytogenetic study prior to and through long-term treatment (Pieńkowska-Grela et al., 2009). On the other hand, in a study by Kaur et al. (2012), six cases (27.2 %) were Ph + ve before treatment and Ph-ve during a follow-up trial (1–3 years of treatment). However, the CML patients in our study (83.0 %) who have normal karyotyping were under therapy, and the result may indicate a good response to treatment. A previous study showed many CML patients with chromosomal abnormalities do not respond well to imatinib therapy. This increases the need for alternative therapeutic options to imatinib to improve treatment outcomes (M Haleem, 2020). According to research, about 90 % of individuals with CML have the Ph chromosome at the time of diagnosis. Karyotyping, a genetic method, assists clinicians in understanding the impact and progression of CML. As a result, specialists recommend this test at the time of diagnosis and at regular intervals throughout the person's treatment (Sampaio et al., 2021).

The study concluded that there was a marked relationship between FokI, ApaI, and Tru91 polymorphisms and the chance of getting leukemia. On the other hand, the polymorphism of Tru91 may not be of particular importance in lymphoma development, as no correlation between lymphoma development and the Tru91 VDR polymorphism was detected. Cytogenetic and molecular testing are essential to the identification of the chromosomal abnormalities in CML patients, and for monitoring response to therapy.

## Declaration of competing interest

The authors declare that they have no known competing financial interests or personal relationships that could have appeared to influence the work reported in this paper.
