# Supplementary figures and images for: The impact of circulating 25-hydroxyvitamin D and vitamin D receptor variation on leukemia-lymphoma outcome: Molecular and cytogenetic study
Source: Saudi J Biol Sci. 2023 Nov 25;31(1):103882. doi: 10.1016/j.sjbs.2023.103882 (PMC10730835; doi:10.1016/j.sjbs.2023.103882)

**Figure S1. VDR gene (Fok1, 265 bp). The gene product was electrophoresed on 1.5% agarose gel**


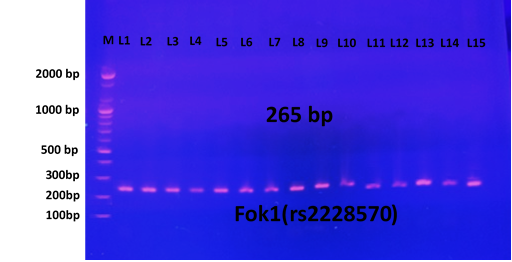

Supplement: Supplementary data 1 [file mmc1.docx]

**Figure S2. VDR gene (Apa1, 740 bp). The gene product was electrophorized on 1.5% agarose gel**


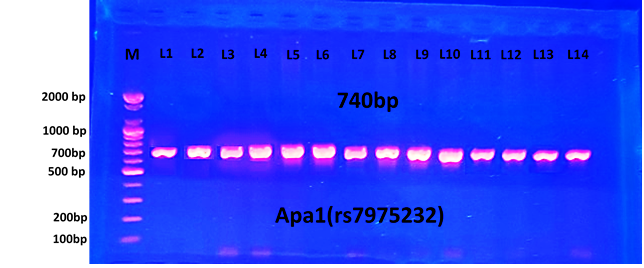

Supplement: Supplementary data 2 [file mmc2.docx]

**Figure S3. VDR gene (Tru91, 331 bp). The gene product was electrophoresed on 1.5% agarose gel**

**
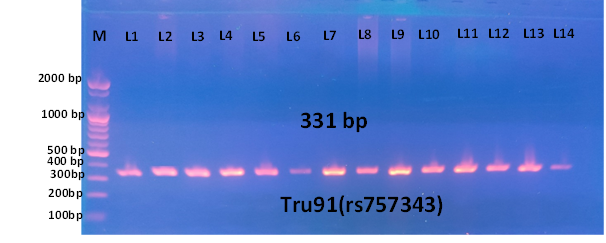
**

Supplement: Supplementary data 3 [file mmc3.docx]
