# Supplementary material for: The impact of circulating 25-hydroxyvitamin D and vitamin D receptor variation on leukemia-lymphoma outcome: Molecular and cytogenetic study
Source: Saudi J Biol Sci. 2023 Nov 25;31(1):103882. doi: 10.1016/j.sjbs.2023.103882 (PMC10730835; doi:10.1016/j.sjbs.2023.103882)
Supplement: Supplementary data 4 [file mmc4.docx]

**Table S1** **Parameters associated with leukemia/lymphoma and control**

| **Parameters** | **CML**  **(n=30)** | **CLL**  **(n=10)** | **HL**  **(n=15)** | **NHL**  **(n =20)** | **Controls**  **(n= 25)** |
| --- | --- | --- | --- | --- | --- |
| **Age** | 41.60 ± 11.746 | 59.80 ± 7.208 | 31.20 ± 10.864 | 51.70 ± 11.859 | 34.72 ± 12.870 |
| **WBC (**×10^9^ /L**)** | 7.107 ± 1.7988 | 26.230 ± 45.559 | 8.133 7.2014 | 6.190 ± 2.3400 | 6.772 ± 1.2249 |
| **Hb (**g/dL**)** | 11.970 ± 1.9148 | 11.970 ± 0.3093 | 11.540 ± 1.7125 | 11.170 ± 1.5332 | 13.632 ± 0.9690 |
| **PLT** (×10^9^ /L**)** | 216.20 ± 65.839 | 140.60± 33.494 | 308.47 ± 104.95 | 244.10 ± 104.63 | 262.24 ± 41.510 |
