# Supplementary material for: The impact of circulating 25-hydroxyvitamin D and vitamin D receptor variation on leukemia-lymphoma outcome: Molecular and cytogenetic study
Source: Saudi J Biol Sci. 2023 Nov 25;31(1):103882. doi: 10.1016/j.sjbs.2023.103882 (PMC10730835; doi:10.1016/j.sjbs.2023.103882)
Supplement: Supplementary data 5 [file mmc5.docx]

**Table S2. The distribution of the study patients based on vitamin D status (severe deficiency, deficiency, insufficiency, or sufficiency).**

| **VD Groups** | **CML**  **No. (%)** | **CLL**  **No. (%)** | **HL**  **No. (%)** | **NHL**  **No. (%)** | **Total**  **No. (%)** |
| --- | --- | --- | --- | --- | --- |
| **Severe deficiency** | 8 (10.6%) | 4 (5.3%) | 9 (12.1%) | 8 (10.6%) | 29 (38.6%) |
| **Deficiency** | 18 (24%) | 6 (8%) | 6 (8%) | 10 (13.3%) | 40 (53.3%) |
| **Insufficiency** | 4 (5.3%) | 0 (0%) | 0 (0%) | 1 (1.3%) | 5 (6.6%) |
| **Sufficiency** | 0 (0%) | 0 (0%) | 0 (0%) | 1 (1.3%) | 1 (1.3%) |
