# Supplementary material for: The impact of circulating 25-hydroxyvitamin D and vitamin D receptor variation on leukemia-lymphoma outcome: Molecular and cytogenetic study
Source: Saudi J Biol Sci. 2023 Nov 25;31(1):103882. doi: 10.1016/j.sjbs.2023.103882 (PMC10730835; doi:10.1016/j.sjbs.2023.103882)
Supplement: Supplementary data 6 [file mmc6.docx]

**Table S3. The distribution of the patients and control groups according to vitamin D status (either deficient or insufficient)**

| **Groups** | **VD Deficiency** | **VD Insufficiency** | **VD Sufficiency** |
| --- | --- | --- | --- |
| **CML No. (%)** | 26 (86.7%) | 4 (13.3%) | 0 (0.0%) |
| **CLL No. (%)** | 10 (100%) | 0 (0.0%) | 0 (0.0%) |
| **HL No. (%)** | 15 (100%) | 0 (0.0%) | 0 (0.0%) |
| **NHL No. (%)** | 18 (90.0%) | 1 (5.0%) | 1 (5.0%) |
| **Control No. (%)** | 5 (20.0%) | 18 (72.0%) | 2 (8.0%) |
