# Supplementary material for: The impact of circulating 25-hydroxyvitamin D and vitamin D receptor variation on leukemia-lymphoma outcome: Molecular and cytogenetic study
Source: Saudi J Biol Sci. 2023 Nov 25;31(1):103882. doi: 10.1016/j.sjbs.2023.103882 (PMC10730835; doi:10.1016/j.sjbs.2023.103882)
Supplement: Supplementary data 7 [file mmc7.docx]

**Table 4S. The cytogenetic aberration, karyotype, and frequency of patients with chronic myeloid leukemia**

| **Cytogenetic aberration** | **Karyotype** | **No. of cases** | **Frequency (%)** |
| --- | --- | --- | --- |
| **Normal Karyotype** | 46, XX  46, XY | 25 | 83.0% |
| **Abnormal Karyotype** | 46, XX, t (9; 22)  46, XY, t (9; 22) | 5 | 17.0% |
